# DPPH‐based Antioxidant Screening and Cellulase‐assisted Hydrodistillation of *Matourea azurea*: Improved Essential Oil Yield and Density Functional Theory Insights Into a Rare Terpene

**DOI:** 10.1002/cbdv.202501109

**Published:** 2025-08-07

**Authors:** Júlio César Gonçalves de Souza, Leiliane do Socorro Sodré de Souza, Ari de Freitas Hidalgo, Guilherme Teixeira de Azevedo, Giovana Lima de Souza, Caroline Dutra Lacerda, Sergio Duvoisin Junior, Anderson Mathias Pereira

**Affiliations:** ^1^ Graduate Program in Environmental Sciences and Sustainability in the Amazon (PPG‐CASA) Federal University of Amazonas Manaus Brazil; ^2^ Department of Animal and Plant Production Federal University of Amazonas Manaus Brazil; ^3^ Department of Chemical Engineering and Food Engineering Federal University of Santa Catarina Florianopolis Brazil; ^4^ Applied Chemistry Research Group for Technology State University of Amazonas Manaus Brazil

**Keywords:** B3LYP, CPCM (water), DPPH, enzymolysis, terpenes

## Abstract

Essential oils are rich in bioactive molecules like terpenes and phenolic compounds, but have low yields, making process efficiency crucial. Enzyme‐assisted hydrodistillation is a sustainable alternative, enhancing oil release by degrading the cell wall. This study applied cellulase enzymolysis before hydrodistillation in *Matourea azurea* (Linden) Colletta; V.C.Souza (*M. azurea*) leaves, testing concentrations of 1.0%, 1.5%, and 2.0%, followed by gas chromatography‐mass spectrometry (GC─MS) analysis to identify chemical alterations. Antioxidant activity was assessed by 2,2‐diphenyl‐1‐picrylhydrazyl (DPPH) scavenging, while density functional theory calculations at B3LYP/6‐311++G(d,p) level evaluated molecular reactivity. The essential oil yielded 0.60%, with a 24% increase at 1% cellulase. GC‐MS confirmed chemical variations while maintaining key compounds (β‐copaen‐4α‐ol, myrtenal, and viridiflorol). The DPPH IC_50_ was 237.70 µg/mL. Computational studies in gas and CPCM (water) showed that 1,4,7‐cycloundecatriene, 1,5,9,9‐tetramethyl‐Z,Z,Z‐ is stable, with lower reactivity in aqueous medium, based on the highest occupied molecular orbital‐lowest unoccupied molecular orbital energy gap. Condensed Fukui indices suggest antioxidant and oxidant potential, corroborated by total and projected density of states analysis, indicating nucleophilic and electrophilic reactive sites. These findings highlight enzyme‐assisted hydrodistillation as an effective strategy to improve essential oil extraction and bioactivity while maintaining sustainable processing.

## Graphical abstract

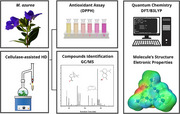

## 1 Introduction

Plants have a high capacity to produce secondary metabolites, estimated at approximately 1 million compounds, which is a significant number compared to other organisms. Among these substances are essential oils, defined as aromatic and volatile compounds produced by plants. These oils consist of a complex mixture of organic compounds, including terpenes, phenols, aldehydes, and esters. In plants, essential oils play crucial roles in defense against herbivores, protection against infections by microorganisms, and attraction of pollinators, aiding in the plant's environmental interaction. In industry, these oils are valuable due to their therapeutic and aromatic properties, being applied in pharmaceutical, cosmetic, and food products for their antimicrobial, antioxidant, and anti‐inflammatory properties [1, 2, 3].

However, essential oils are present in small amounts in plants, being produced mainly in specific tissues, such as the glandular trichomes found in seeds, flowers, branches, and roots, in relatively low proportions compared to the plant's total dry mass. Their extraction by more traditional methods, such as hydrodistillation, primarily involves physical processes like heating, vaporization, condensation, and separation by density. The plant material is placed in water or a solvent, and the mixture is heated. This heating generates vapor, which serves as a medium to transport the volatile compounds from the plant cells. As the temperature increases, water vapor penetrates the plant cells, breaking cellular structures and releasing the essential oils. These volatile compounds are carried out of the plant by vapor [4].

The plant cell wall has significant thermal resistance due to its structural composition, which includes cellulose, hemicellulose, and lignin. These components form a rigid and durable matrix, protecting plant cells from extreme temperature variations. Cellulose, a glucose polymer, provides rigidity and mechanical strength, while hemicellulose and lignin increase the cell wall's thermal stability and impermeability. Among various strategies to enhance extraction efficiency and preserve compounds, enzymolysis is particularly notable. The use of enzymolysis in essential oil extraction offers several advantages, particularly in terms of efficiency and compound preservation, as it facilitates the release of volatile compounds without the need for high temperatures [5, 6].

The application of enzymes in hydrodistillation directly contributes to the Sustainable Development Goals, a recurring societal concern considering climate change and social crises. In this context, SDG 9 (Industry, Innovation, and Infrastructure) and SDG 12 (Responsible Consumption and Production) are addressed by promoting a more efficient extraction of bioactive compounds from plants, using less energy and eliminating the need for harsh chemical solvents such as hexane and n‐hexane. This approach represents a significant advancement in the natural products industry [7]. Enzymes, such as cellulase, effectively break down plant cell walls, enabling the extraction of high‐quality essential oils with a lower environmental impact. This innovation pushes the industry toward cleaner and more technological practices, fostering the creation of sustainable and competitive products. Additionally, enzyme‐assisted hydrodistillation saves resources and minimizes waste production, contributing to a more responsible production cycle. Thus, this technique demonstrates how biotechnology can transform industrial processes, promoting innovative and sustainable practices that meet the growing market demand for high‐quality products with low environmental impact [8].

The use of enzymes in essential oil extraction processes can also lead to the generation of new molecules through biotransformation reactions. These enzymes not only facilitate the breakdown of cell walls but can also transform compounds into new derivatives through reactions such as hydrolysis, oxidation, and isomerization, depending on the extraction conditions and the type of enzyme used. In this scenario, density functional theory (DFT) is a powerful tool for investigating the electronic structure of these generated molecules and predicting their chemical and physical properties. The use of DFT allows for the analysis of molecular reactivity and the study of non‐covalent interactions, providing crucial information about the antioxidant potential, cytotoxicity, and other biological activities of derived compounds. This information guides the development of compounds with pharmaceutical potential [9]. Thus, the integrated use of enzymes and DFT analysis directly contributes to advances in health and well‐being, aligning with SDG 3 by enabling sustainable innovations in health products and natural therapies [10, 11].

The species chosen for this research was *M. azurea*. Most available studies in literature mention its synonyms *Achetaria azurea* (Linden) V.C. Souza or *Otacanthus azureus* (Linden) Ronse. Belonging to the Plantaginaceae family, it is naturally found in the southeastern region of Brazil. However, due to its high adaptability, it has been introduced to the northern region and is cultivated in various locations worldwide, including the United States, France, India, and Japan. It exhibits heliophilic behavior, high water demand, and easy propagation through asexual reproduction. Its essential oil is rich in mono‐ and sesquiterpenes, characterized mainly by molecules such as β‐copaen‐4α‐ol and myrtenal. Additionally, studies report its antifungal effects and inhibitory activity against *Leishmania amazonensis* [12, 13, 14].

Given this context, this research aimed to compare traditional hydrodistillation with enzyme‐assisted hydrodistillation, evaluating the methods in terms of yield and chemical composition of the extracted essential oils. The investigation also included analyzing the antioxidant activity of the crude essential oil using the 2,2‐diphenyl‐1‐picrylhydrazyl (DPPH) method to assess its potential for neutralizing free radicals. Furthermore, the research applied DFT to study the electronic structure and chemical properties of 1,4,7‐cycloundecatriene, 1,5,9,9‐tetramethyl‐Z,Z,Z‐, a molecule resulting from enzymolysis of *M. azurea* leaves. Molecular and electronic properties, Fukui functions, molecular electrostatic potential (MEP) surface values were calculated. Reduced density gradient (RDG) and non‐covalent interactions (NCI) analyses were also performed to describe weak molecular interactions. Finally, total density of states (TDOS) and fragment analyses based on Fukui results were conducted to measure energy and distribution around the molecule's orbitals. Thus, the study aims to provide a foundation for the more efficient and sustainable use of enzymatic methods in essential oil extraction and explore the bioactive potential of the derivatives produced.

## 2 Results and Discussion

### 2.1 Cellulase‐Assisted Hydrodistillation

**FIGURE 1:**
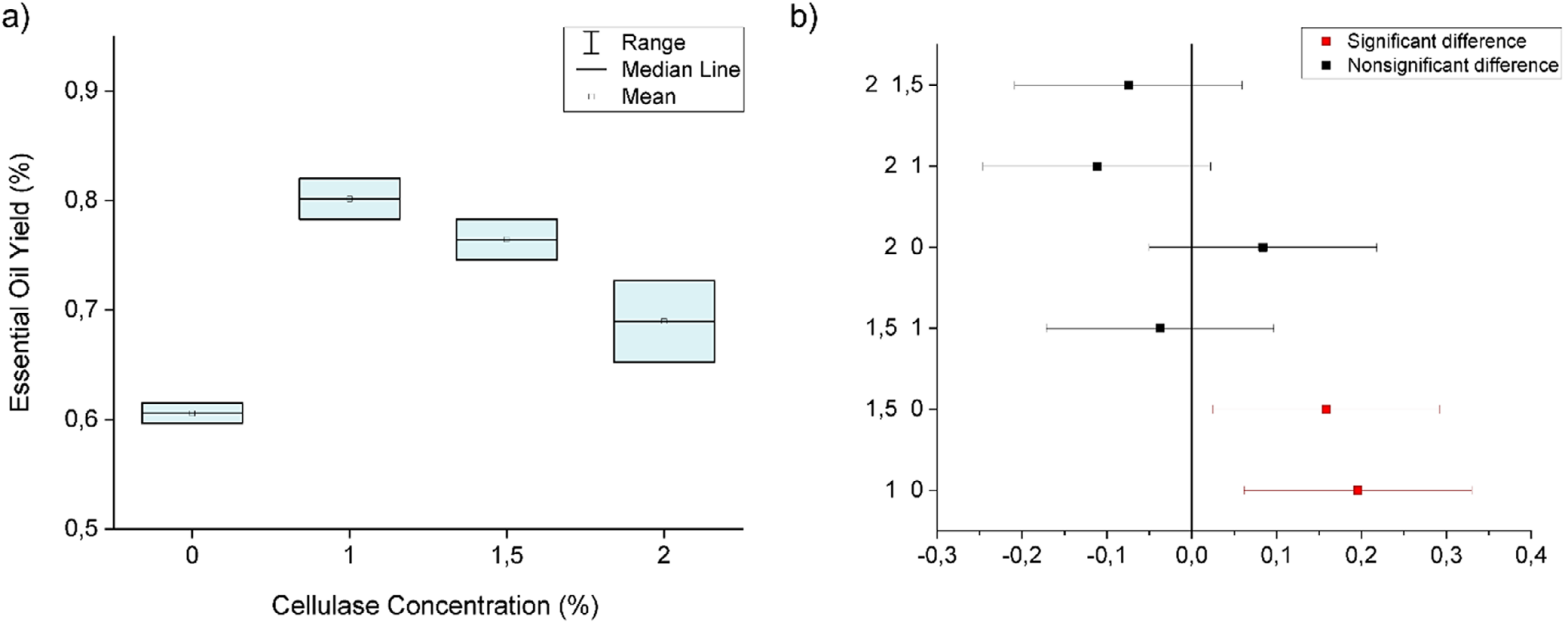
(a) Cellulase‐assisted hydrodistillation. (b) Post‐hoc – Tukey test (cellulase).

The essential oil of *M. azurea* has a yellowish color reminiscent of citrine and a density of 1.05 g/mL. The leaves have an average moisture content of 24.90% ± 0.47. The mean yield (grams/dry basis) for traditional hydrodistillation (control) was 0.61% ± 0.01. For the enzymolysis‐pretreated trials, the yields at different cellulase concentrations were as follows: 1% (0.80% ± 0.03), 1.5% (0.76% ± 0.03), and 2% (0.69% ± 0.05). The following Figure 1a illustrates the extraction volumes quantitatively, showing an increase in the absolute yields of *M. azurea* essential oil with cellulase‐assisted hydrodistillation. It is evident that the statistical differences occur between the treatments with 1% and 1.5% cellulase compared to the control, with increases of 32.31% and 26.16%, respectively. The 2% concentration showed an increase of 13.85%. According to the data, the optimal cellulase concentration is 1%. Comparable results were reported for *Elettaria cardamomum* seeds, in which the optimal conditions for cellulase‐assisted enzymolysis were similar to those established in the present study, with slight variations such as a longer incubation time (90 min), attributed to the characteristics of the substrate. In this case, the optimal enzyme concentration of 1% led to a 10% increase in essential oil yield, a value lower than that obtained from *M. azurea* leaves, likely due to their lower lignin content and the resulting influence on extraction methods [8].

The analysis of variance (ANOVA) results in Table 1 show a *p*‐value less than 0.05, indicating that at least one treatment is statistically different. Tukey test plots are displayed in Figure 1b.

**TABLE 1:** Analysis of variance (ANOVA) one‐way – cellulase‐assisted hydrodistillation of the essential oil of the *M. azurea* leaves.

Source of variation	DF^a^	SS^b^	MS^c^	*F*‐value	Prob > *F*	R^2^	Coefficient of variation
**Model**	3	0.04497	0.01499	13.82393	0.01408	0.91203	0.04603
**Error**	4	0.00434	0.00108				
**Total**	7	0.04931					

^[a]^ Degree of Freedom.^[b]^ Sum of Squares.^[c]^ Mean of Squares

When the confidence interval does not intersect the zero line, it indicates a statistically significant difference (highlighted in red). The position of the mean on the horizontal axis reveals whether the mean of the first treatment in the comparison is higher or lower. For example, when comparing the 2% and 1.5% treatments, there is no statistical difference since the interval intersects the zero line, and the position in the negative region suggests that the mean essential oil volume extracted with 2% cellulase was lower than that extracted with 1.5%.

The ANOVA – One‐Way in Table 1 presents an *R* ^2^ value of 0.91, confirming that the model effectively captured the variability in the data based on the analyzed factors. This parameter ranges between 0 and 1, with values closer to 1 indicating a stronger explanatory capacity for the sources of variation.

Degrees of freedom (DF), sum of squares (SS), and mean square (MS) are key components in ANOVA, measuring the total variation in the data and partitioning it between and within groups. DF indicates the number of values free to vary, while SS quantifies the total variation. MS, obtained by dividing SS by DF, is used to calculate the F‐test, which determines whether the differences between groups are statistically significant [15, 16]. The plant cell wall is composed of polysaccharides, proteins, phenolic compounds, and mineral salts, with cellulose being the most abundant polysaccharide in nature.

**FIGURE 2:**
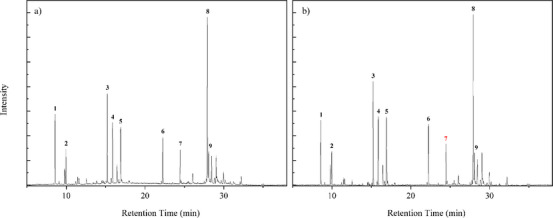
Total ion chromatogram (TIC) of the essential oil from *M. azurea*. (a) Control, with corresponding peaks and compounds: 1 ‐ *Cyclofenchene*, 2 ‐ *β‐Pinene*, 3 ‐ *Pinocarveol*, 4 ‐ *Pinocarvone*, 5 ‐ *Myrtena*l, 6 ‐ *Copaene*, 7 ‐ *Humulene*, 8 ‐ *β‐Copaen‐4α‐ol*, 9 ‐ *Viridiflorol*. (b) Sample subjected to enzymatic pretreatment with 1% cellulase, with corresponding peaks and compounds: 1 ‐ *Cyclofenchene*, 2 ‐ *β‐Pinene*, 3 ‐ *trans‐Pinocarveol*, 4 ‐ *Pinocarvone*, 5 ‐ *Myrtenal*, 6 ‐ *Copaene*, 7 ‐ *1,4,7‐Cycloundecatriene, 1,5,9,9‐tetramethyl‐Z,Z,Z*, 8 ‐ *β‐Copaen‐4α‐ol*, and 9 ‐ *Viridiflorol*.

**FIGURE 3:**
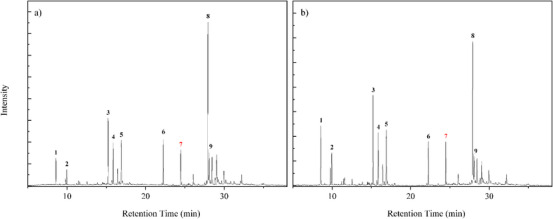
Total ion chromatogram (TIC) of the essential oil from *M. azurea*. (a) Sample subjected to pretreatment with 1.5% cellulase. The peaks and their corresponding compounds are: 1 ‐ *trans‐β‐Ocimene*, 2 ‐ *β‐Pinene*, 3 ‐ *Pinocarveol*, 4 ‐ *Pinocarvone*, 5 ‐ *Myrtena*l, 6 ‐ *Copaene*, 7 ‐ *1,4,7‐Cycloundecatriene, 1,5,9,9‐tetramethyl‐Z,Z,Z, 8 ‐ β‐Copaen‐4α‐ol*, 9 ‐ *Viridiflorol*. (b) Sample subjected to enzymatic pretreatment with 2% cellulase, with corresponding peaks and compounds: 1 ‐ *Cyclofenchene*, 2 ‐ β‐Pinene, 3 ‐ trans‐Pinocarveol, 4 ‐ Pinocarvone, 5 ‐ Myrtenal, 6 ‐ Copaene, 7 ‐ *1,4,7‐Cycloundecatriene, 1,5,9,9‐tetramethyl‐Z,Z,Z*, 8 ‐ *β‐Copaen‐4α‐ol*, and 9 ‐ *Viridiflorol*.

The GC/MS analysis identified the chemical profile of *M. azurea* essential oil. A qualitative difference in the oil composition was observed after enzymatic treatment with Celluclast 1.5 L, as shown in Table 2. Among the notable changes were the appearance of 1,5,9,9‐tetramethyl‐Z,Z,Z‐ in all concentrations and p‐mentha‐2,8‐dien‐1‐ol at a 1% concentration. Figures 2 and 3 present the chromatograms of the essential oils obtained from each concentration of cellulase‐assisted distillation. The molecule β‐Copaen‐4α‐ol (20.73%) was the major compound in the essential oil from the control sample, followed by pinocarveol (9.49%), myrtenal (8.74%), pinocarvone (6.31%), and cyclophenene (6.30%). Overall, these compounds maintained their relative percentages across the different enzyme concentrations. Peak 7, highlighted in red, marks the peak identifying the terpene 1,4,7‐Cycloundecatriene, 1,5,9,9‐tetramethyl‐Z,Z,Z in chromatograms where it was done the hydrodistillation‐assisted by cellulase assay was performed.

**TABLE 2:** Chemical composition of *M. azurea* essential oil.

Name	RI	RT	Control	1%	1.5%	2%
trans‐β‐Ocimene	976	8.576	—	—	2.82	—
Cyclofenchene	729	8.574	6.3	5.36	—	5.88
Camphene	943	9.081	0.22	0.19		0.14
Sabinene	897	9.800	1.52	1.79	0.73	1.74
(−)‐β‐Pinene	943	9.955	3.24	2.98	1.74	3.25
Terpinolene	1052	11.213	0.31	0.23		—
β‐Cymene	1042	11.448	0.75	0.73	0.53	0.69
D‐Limonene	1018	11.607	0.68	0.57	0.42	0.78
γ‐Terpinene	998	12.542	0.54	0.56	0.47	0.67
Linalool	1082	13.864	0.22	0.14	0.28	0.36
Dehydro sabinene ketone	983	14.502	0.31	0.36	0.31	0.38
trans‐p‐Menth‐2‐ene‐1‐ol	1109	14.596		0.37		
trans‐Pinocarveol	1131	15.212	9.49	10.02‐	8.17	10.22
p‐Mentha‐2,8‐dien‐1‐ol	1140	15.224	—	0.29	—	—
Pinocarvone	1114	15.870	6.31	6.77	5.11	6.06
(−)‐Terpinen‐4‐ol	1137	16.447	2.41	2.35	2.31	2.86
trans‐p‐mentha‐1(7),8‐dien‐2‐ol	1201	16.678		0.60		
(1R)‐(−)‐ Myrtenal	1136	16.909	8.74	9.91	8.24	9.42
(+)‐Cyclosativene	1125	22.075	0.29	0.31	0.25	—
Copaene	1221	22.258	4.95	5.84	5.47	4.93
Caryophyllene	1494	23.504	0.16	0.18		0.17
Humulene	1579	24.466	3.80	—	—	—
**C11‐TMTb**	1579	24.475	—	4.2	4.58	5.22
Alloaromadendrene	1386	24.591	0.32	0.39	0.41	0.33
Cadina‐1(10),4‐diene	1469	26.067	1.45	1.54	1.79	1.57
β‐Copaen‐4α‐ol	1405	27.920	20.73	20.17	23.93	19.09
Caryophyllene oxide	1507	27.720	0.67	0.59	0.60	0.56
Viridiflorol	1530	28.084	4.6	4.22	5.11	4.28
Ylangenal	1410	29.620	0.34	0.24	0.39	0.30

^[a]^Retention Index. ^[b]^Cycloundecatriene, 1,5,9,9‐tetramethyl‐ Z,Z,Z‐.

Structurally, cellulose is made up of long, linear chains of β‐D‐glucopyranose units linked by β (1→4) bonds, which make it resistant to many solvents, including water. The enzyme Celluclast 1.5 L hydrolyzes these β‐D‐glycosidic bonds in cellulose and other types of β‐D‐glucans. Although cellulose itself is insoluble, enzymatic digestion products, such as cellobiose and sugar monomers, are water‐soluble. Enzymatic hydrolysis in aqueous media offers advantages, as pure water alone takes longer to degrade the cell wall of tissues that store oily compounds, thereby reducing efficiency and yield [7, 17].

Enzymatic hydrolysis in aqueous media offers advantages, as pure water alone takes longer to degrade the cell wall of tissues that store oily compounds, thereby reducing efficiency and yield [7, 17].

The enzymatic hydrolysis of cellulose involves the synergistic action of endoglucanase, exoglucanase (or cellobiohydrolase), and beta‐glucosidase. Endoglucanases randomly hydrolyze accessible intramolecular β‐1,4‐glycosidic bonds in cellulose chains, producing new chain ends. Exoglucanases gradually cleave cellulose chains at their ends to release soluble cellobiose or glucose. β‐Glucosidases then hydrolyze cellobiose into glucose, eliminating cellobiose inhibition. The combined action of endoglucanases and exoglucanases modifies the molecule's topography, increasing the rate of hydrolysis [17]. In this work [18], using Celluclast 1.5 L, it was demonstrated that its action resulted in the production of three main sugars: xylose, glucose, and a small amount of cellobiose, along with galactose and arabinose.

In Figure 1a, it is observed that all concentrations used resulted in an increase in essential oil yield. However, as concentration increases, there is a decrease in the efficiency of enzymatic digestion. Since the data do not follow Michaelis‐Menten kinetics, the possibility of enzymatic saturation is excluded. In this scenario, with a constant substrate volume, the curve would display a plateau after reaching the optimal concentration, forming the shape of a rectangular hyperbola [19]. This behavior can be explained by enzymatic inhibition caused by the degradation products of cellulose, such as cellobiose and glucose, or by interactions with aromatic substances like terpenes and phenols from the biomass. Non‐productive binding of the enzyme to lignin may also occur, potentially due to the type of protein or allosteric modifications [20].

Enzymatic inhibition can occur in two primary forms: allosteric and competitive, each affecting enzyme function in distinct ways. In competitive inhibition, the inhibitor directly competes with the substrate for binding to the enzyme's active site, reducing the rate of enzyme‐substrate complex formation. In contrast, allosteric inhibition occurs when the inhibitor binds to a site other than the active site, known as the allosteric site, inducing a conformational change that decreases the enzyme's affinity for the substrate or its catalytic activity.

As the concentration of reactants increases, the reaction rate accelerates, and inhibitory products form more quickly, compromising the efficiency of digestion to the point where no statistically significant difference is observed between the 2% concentration and the control [21, 22, 23].

The variation in composition with enzyme‐assisted extraction concentrations may be attributed to the interaction between terpenes and the products of enzymolysis. This includes the solubility of cellobiose and sugar monomers, which may release ions into the solution and facilitate oxidation reactions. Alternatively, reducing sugars might act as antioxidants. Additionally, the solubilization of preservatives and stabilizers in the enzymatic complex, such as potassium sorbate and sodium chloride, could contribute to the formation of new products in varying proportions [24, 25, 26].

Recent studies have supported the effectiveness of enzymes and biological treatments as strategies to optimize essential oil extraction, improving both yield and efficiency. It was observed that the enzymatic extraction of *Cinnamomum longepaniculatum* increased the number of oxygenated compounds and oil yield by up to 53.55% when β‐glucan and hemicellulase were used together [27]. Similarly, another research reported that using Viscozyme in the extraction of vetiver (*Vetiveria zizanioides*) essential oil increased the yield by 23% and reduced the extraction time by 10 hours without significantly altering the oil's qualitative profile [28]. In another study, it was observed that the combination of cellulase and hemicellulase increased the levels of linalool, lavandulyl acetate, and α‐terpineol in the essential oil of *Lavandula angustifolia* [29]. Additionally, the application of submerged fermentation as a pretreatment for *Melaleuca leucadendra*, using the biological agents *Trichoderma viride* and *Phanerochaete chrysosporium*, resulted in a yield increase of up to 3.47% and facilitated extraction by loosening the lignocellulosic structure of the leaves [30].

These studies reinforce that the use of enzymes and biological agents can improve the yield, efficiency, and composition of essential oils, demonstrating the feasibility of this method for various species, including *M. azurea*.

### 2.2 Antioxidant Activity (DPPH)

The DPPH inhibition percentage of the conventional hydrodistillation essential oil increased proportionally with the concentration, and based on the R^2^ value, it can be stated that the reduction occurs in a dependent and linear manner. In the DPPH graph (Supporting Information), it is possible to see that the highest inhibition percentage occurred at a concentration of 300 µL/mL, reaching 62.60%. The IC_50_ calculated from the equation of the linear regression was 225.41 µL/mL (237.70 µg/mL). The moderate DPPH radical scavenging capacity observed in this study becomes evident when compared to previous findings in the literature, including both other plant species and standard antioxidant compounds such as butylated hydroxyanisole (BHA) and ascorbic acid, which require much lower concentrations for inhibition. For instance, the essential oil of *Artemisia herba‐alba* exhibits an IC_50_ of 5.038 µg/mL, while in the present experiment, ascorbic acid, used as a positive control, displayed an IC_50_ of 3.65 µg/mL. In another study, BHA showed an IC_50_ of only 160 µg/mL, whereas the sample evaluated in this work presented an IC_50_ of 237.70 µg/mL, indicating a lower antioxidant activity than the synthetic standard, yet still significant. Compared to the essential oil of clove (*Syzygium aromaticum*), which has an IC_50_ of 320 µg/mL, the result obtained here demonstrates greater antioxidant efficiency. However, it remains considerably less potent than essential oils such as that of thyme (*Thymus vulgaris*), with an IC_50_ of 400 µg/mL, suggesting that the species evaluated in this study exhibits an intermediate antioxidant effect within the spectrum of essential oils reported in the literature [31, 32].

Free radicals are molecules that possess one or more unpaired electrons in their valence shell, resulting in instability, short lifespan, and high reactivity. These radicals can be generated through contact with external sources such as radiation, pollutants, drugs, cosmetics, and other agents. In the human body, they act as regulators of homeostasis, playing a dual role in biological systems: they can be toxic to aerobic metabolism, causing oxidative damage and tissue dysfunction, which, over time, may lead to tumor formation and aging‐related diseases. Conversely, they also participate in cellular signaling, activating beneficial stress responses [33, 34, 35].

In the literature, there is a study where methanol was used as a solvent, a polar substance that, according to studies based on Density Functional Theory, reduces the dissociation energy of the O‐H bond, favoring hydrogen atom transfer [36]. However, other researchers argue that the process occurs through the transfer of a single hydrogen electron [37]. Both mechanisms are considered possible since increased polarity in the medium reduces vertical ionization potential (VIP) values, facilitating hydrogen electron transfer [36]. Historically, the reaction mechanism between phenolic antioxidants and peroxyl radicals was attributed to hydrogen atom transfer. Still, recent studies show that it can also occur via proton‐coupled electron transfer. Furthermore, compounds with aldehyde functional groups can neutralize peroxyl radicals similarly. The essential oil of the species *M. azurea*, analyzed in this study, showed moderate capacity to reduce DPPH to DPPH‐H even at high concentrations. This was evident as the solution retained a light violet coloration instead of turning yellow as expected at the end of the experiment.

On the other hand, in a parallel study, the same species was evaluated using a DPPH assay dissolved in methanol, achieving a 90% scavenging capacity at a concentration of 1 mg/mL, with an IC_50_ of 88.5 µg/mL [38]. However, these results were achieved using essential oil obtained through different methods, including grinding the plant material to a pasty state, extracting the oil by steam distillation, and separating it using diethyl ether, rather than conventional hydrodistillation.

An analysis conducted with the essential oil of 32 plant species using principal component analysis grouped the species based on antioxidant capacity against DPPH and correlated the results with their chemical constituents [37]. Phenolic compounds showed a significant explanatory weight for lower IC_50_ values, while the results suggested a slight positive synergistic effect between phenols and alcohols. Using response surface methodology, a predictive model for IC_50_ was generated, in which aldehydes had little influence, followed by alcohols, phenols, and sesquiterpenes. This indicates that aldehydes, such as myrtenal, may have low reactivity with DPPH. However, grouping functional groups without considering their chemical structures or reaction mechanisms can lead to underestimated model interpretations [38]. Myrtenal, an aldehyde found in high concentrations in *M. azurea* essential oil, is reported as a strong antioxidant, demonstrating a high scavenging capacity for hydroxyl radicals, superoxide anions, and nitric oxide in a concentration‐dependent way [36]. Viridiflorol, another isolated molecule, also exhibits DPPH scavenging capacity, with an IC_50_ of 74.7 µg/mL, compared to ascorbic acid, which has an IC_50_ of 21.66 µg/mL [39].

Although a correlation between chemical structure and antioxidant capacity is expected, in practice, this relationship is not always linear. The position of antioxidant groups in the molecule can influence electronic configurations, either favoring or hindering electron donation. Despite its widespread use, the DPPH method has significant limitations, such as the absence of an oxidant substrate, which compromises its applicability to biological systems. Additionally, the method does not assess enzyme‐mediated redox mechanisms, underestimating its relevance in biological studies [40].

## 3 Density Functional Theory

### 3.1 The Frontier Orbitals and Quantum Descriptors

Studies on the components of essential oils using experimental and theoretical methods play a crucial role in understanding their impacts and potential applications. The highest occupied molecular orbital (HOMO) and the lowest unoccupied molecular orbital (LUMO) are the key orbitals involved in chemical stability [41].

**FIGURE 4:**
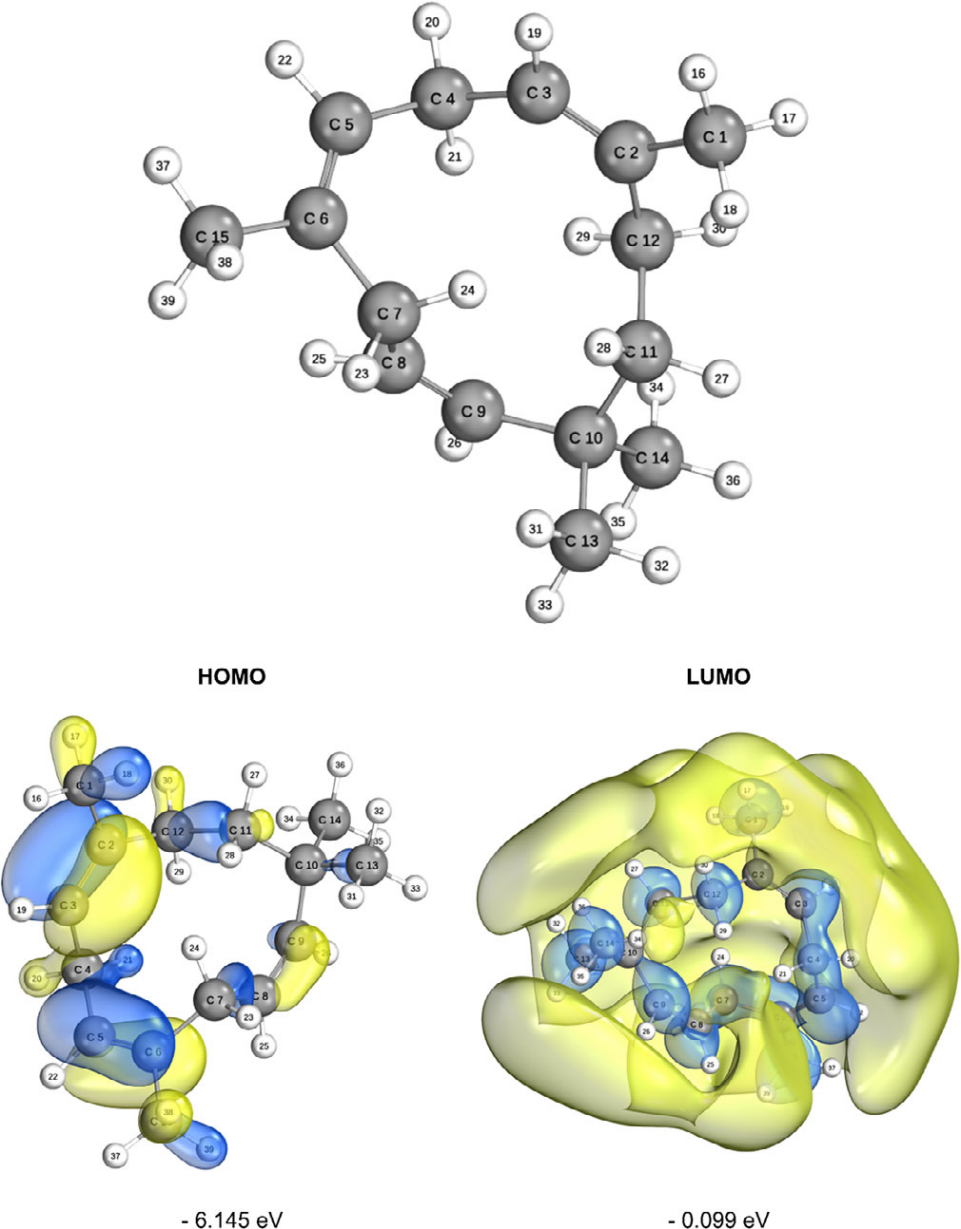
Representation of the optimized molecular structure and frontier molecular orbitals (the highest occupied molecular orbital [HOMO] and the lowest unoccupied molecular orbital [LUMO]) of the studied compound. The energies of the HOMO and LUMO orbitals are indicated in eV. The calculations were performed using the B3LYP functional, and the visualizations were generated using IboView software.

These data are presented in Figure 4 alongside the molecular geometry optimization under implicit solvation in an aqueous medium. Calculations were performed for the Gap_HOMO‐LUMO_, chemical potential, electronegativity, global hardness and softness, electrophilicity index, ionization potential, nucleophilicity index, electron affinity (EA), maximum charge transfer index, and reduction potential, as compiled in Table 3. The lobes of the frontier orbitals (HOMO and LUMO) can be interpreted in terms of color, size, and position. The colors represent the phase of the orbital, indicating the sign of the wave function in different regions, which should not be confused with electric charge. Interactions between regions of the same color across molecules result in constructive interference, while opposing colors indicate destructive interference. The phase is crucial for determining the type of interaction that occurs during bond formation, such as bonding or antibonding interactions [42, 43]. The size of the frontier orbital lobes reflects the electron density in a specific region of the molecule; larger lobes correspond to a higher probability of electron presence. In the HOMO, this indicates a greater tendency to donate electrons, whereas in the LUMO, larger lobes signify areas more likely to accept electrons [44]. Electron density is thus directly related to molecular reactivity, with dense regions in the HOMO or accessible areas in the LUMO being more reactive. The position of the lobes reveals the atoms or functional groups involved in the interactions.

**TABLE 3:** Quantum descriptors calculated from frontier orbital energies.

Descriptor	Gas (eV)	Water (eV)
GapHOMO‐LUMO (ΔE)	5.937	6.244
Chemical potential (μ)	−3.1905	3.023
Electronegativity (χ)	3.1905	3.122
Global hardness (η)	2.9685	3.122
Global softness (S)	0.337	0.3203
Electrophilicity index (ω)	1.715	2.927
Ionization potential (IP)	6.159	6.145
Nucleophilicity index (N)	6.159	0.099
Electron affinity (EA)	0.222	−0.968
Max. charge transfer index (ΔN_max_)	1.075	6.244

Molecule reactivity calculated in B3LYP/6‐311++G(d,p) level.

For the HOMO, this highlights nucleophilic regions of the molecule, while for the LUMO, it identifies electrophilic regions. The molecular frontier energy, or “Gap_HOMO‐LUMO_”, is the energy difference between HOMO and LUMO. A smaller Gap_HOMO‐LUMO_ value generally indicates higher chemical reactivity, as a lower energy difference between these orbitals facilitates electronic transitions, making the molecule more reactive [42]. In oxidation reactions, smaller molecular Gap_HOMO‐LUMO_ energy improves antioxidant performance [45]. In this study, the Gap_HOMO‐LUMO_ value in the gas phase was calculated as 5.937 eV, while in the aqueous phase, it was 6.046 eV, indicating that the molecule exhibits lower reactivity in the aqueous medium due to the increase in the energy gap. When the molecular Gap_HOMO‐LUMO_ decreases, the reactivity and instability of the molecule increase [41]. It is important to note that “binding capacity” is not solely a function of HOMO and LUMO energies; other factors, such as molecular geometry and the nature of the orbitals involved in the interaction, also play a significant role in reactivity and chemical bond formation [46].

The EA, related to the LUMO, represents the energy released when capturing an electron, while the ionization potential (IP) associated with the HOMO indicates the energy required to remove an electron. In the presented data, the EA was calculated as 0.222 eV in the gas phase and 0.099 eV in the aqueous phase, indicating a lower tendency for electron capture in the aqueous medium. Meanwhile, the IP was 6.159 eV in the gas phase and 6.145 eV in the aqueous phase, suggesting a slight reduction in the energy required for electron removal in the aqueous medium [45, 47, 48]. Electronegativity has a direct relationship with electrophilicity, while nucleophilicity shows an inverse relationship with it [49, 50]. According to the data presented, in the gas phase, the electronegativity (χ) was calculated as 3.191 eV, and in the aqueous phase, it was 3.122 eV, reflecting a slight decrease in the aqueous medium. Electrophilicity (ω), which also depends on electronegativity, was 1.715 eV in the gas phase and 1.612 eV in the aqueous phase, indicating a lower electrophilic tendency in the aqueous medium. On the other hand, nucleophilicity (N) was 6.159 eV in the gas phase and 0.620 eV in the aqueous phase, demonstrating an inverse relationship, with greater nucleophilic capacity in the gas phase. These values show that electronegativity and related descriptors are strongly influenced by multiple factors, including electronic structure, orbital configuration, and the system's environment, such as pressure and the solvent medium [41, 45].

### 3.2 Mapping of ELF, LOL and Electrostatic Potential

The electron localization function (ELF) is an essential tool in theoretical and computational chemistry, developed to better understand the organization and distribution of electrons in molecules and solids. This function allows for visualizing regions where electrons are more concentrated, such as in covalent bonds and lone pairs, facilitating the analysis and characterization of different chemical interactions. The localized orbital locator (LOL) is a function designed to map the localization of electrons in a molecule or solid, similarly to ELF. LOL is based on the electronic current density and helps identify regions where electrons are highly localized, especially around atomic nuclei and bonding areas between atoms. While ELF is based on the electron pair localization theory (utilizing the concept of Fermi pairs), LOL derives from the analysis of the electronic current density associated with localized orbitals. ELF is calculated from the Pauli correlation function, whereas LOL is calculated based on the local electronic kinetics, using a function that represents the localization efficiency in specific regions [48, 51, 52, 53].

**FIGURE 5:**
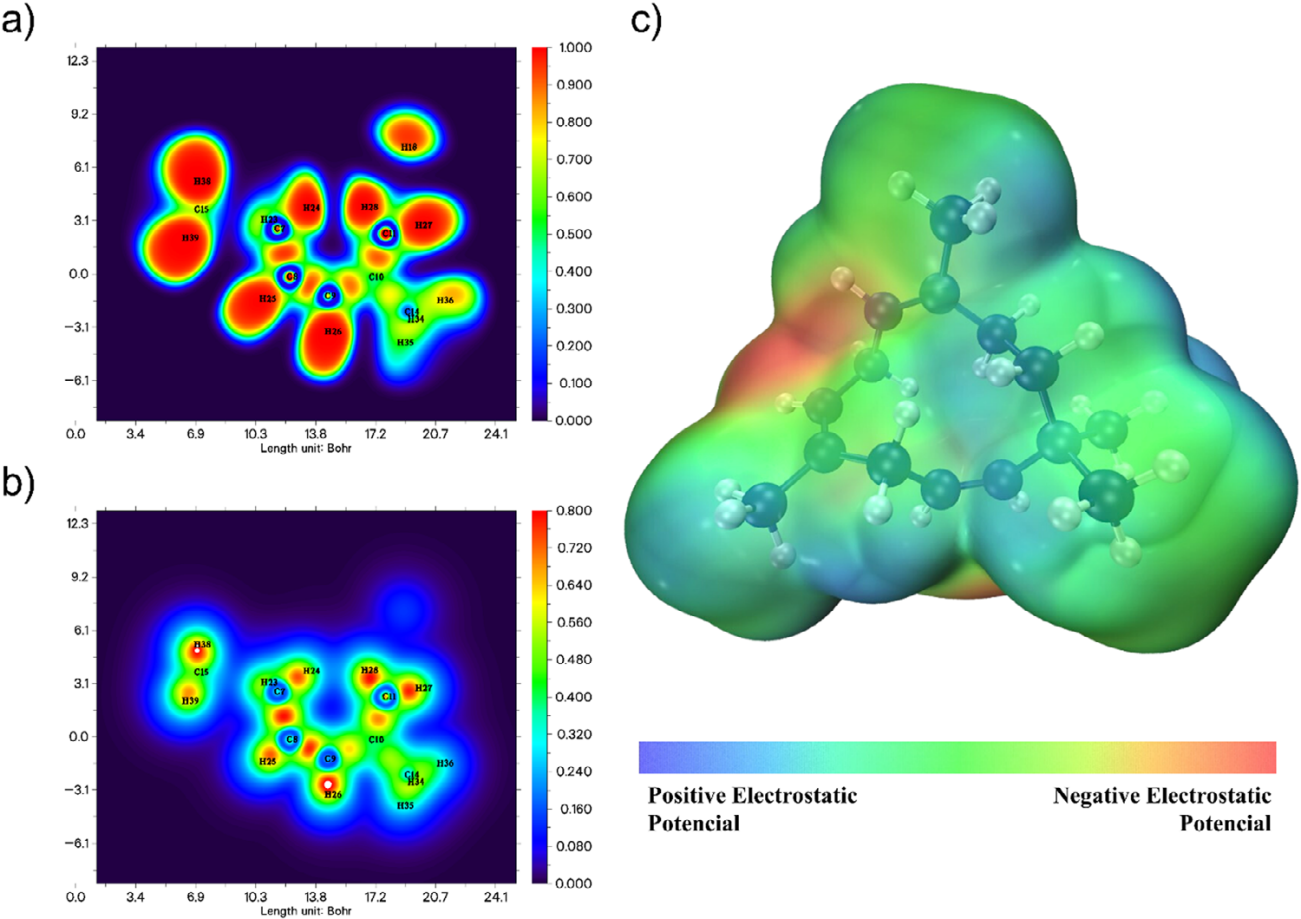
(a) Electron localization function (ELF), (b) localized orbital locator (LOL), and (c) molecular electrostatic potential (MEP) of 1,4,7‐cycloundecatriene, 1,5,9,9‐tetramethyl‐Z,Z,Z‐. The MEP isosurface value for the is 0.001 a.u.

The MEP is a visual representation of the electrostatic potential generated by a molecule in the surrounding space. This map illustrates how the partial charges of atoms and electronic distribution affect the electrostatic environment near the molecule [9]. Figure 5 below illustrates these parameters. The MEP shows relative symmetry between red and blue areas, suggesting a molecule with well‐defined poles and the potential to interact specifically and predictably with other molecules. This characteristic can be relevant for understanding reactivity and intermolecular behavior, particularly in contexts of solvation, protein binding, or supramolecular complex formation. Such an organization is common in molecules capable of forming directed electrostatic interactions, such as hydrogen bonds or dipole‐dipole interactions, as the charge separation facilitates these interactions. In the Supporting Information is available the MEP with an isovalue of 0.002 a.u. where it is noticeable that the molecule has a homogeneous potential electric distribution. This suggests that hydrocarbons may have a good interaction with the cellular membrane [54, 55, 56, 57].

### 3.3 Condensed Fukui functions

The condensed Fukui functions are used to characterize the regioselectivity of an atom ‘r’ in a molecule. For this purpose, Fukui functions were calculated using Multiwfn, with atomic charges obtained through Hirshfeld analysis. The Fukui function for electrophilic attack (f^−^_r_) is determined by the difference between the atomic charge in the neutral system and the cationic system. The Fukui function for nucleophilic attack (f⁺_r_) is obtained by the difference between the atomic charge in the anionic system and the neutral system. The Fukui function for radical attack (f⁰_r_) is the average of the differences in atomic charges between the anionic and cationic systems.

Additionally, the fΔ_r_ index, which shows the difference between the propensity for nucleophilic and electrophilic attacks at a specific site, is calculated as the difference between the functions, where positive results are interpreted as nucleophilic attack regions and negative results as electrophilic attack regions [41, 51, 58].

Table 4 compiles these indices for each atom in the molecule. Atoms 2C, 5C, 3C, 22H, and 20H constitute a region more susceptible to nucleophilic attack based on the descending order of the Fukui delta. Similarly, atoms 18H, 38H, 23H, 7C, and 28H are more prone to electrophilic attack. Interestingly, 2C and 5C form double bonds, where the π bond increases the propensity for participation in chemical reactions compared to σ bonds and contributes more to electron density. These atoms are close to regions of high electronegativity, as observed in the MEP.

**TABLE 4:** Condensed Fukui functions of 1,4,7‐Cycloundecatriene, 1,5,9,9‐tetramethyl‐Z,Z,Z‐.

Number	Atoms	f^+^_r_	f‐r	f0r	f^Δ^_r_
1	C	−0.082138	−0.024669	−0.106807	−0.057469
2	C	−0.016939	−0.102402	−0.119341	0.085463
3	C	−0.047661	−0.101847	−0.149508	0.054186
4	C	−0.001339	−0.022799	−0.024138	0.021460
5	C	−0.022755	−0.092454	−0.115209	0.069699
6	C	−0.057485	−0.089854	−0.147339	0.032369
7	C	−0.088490	−0.018050	−0.106540	−0.070440
8	C	−0.002641	−0.011006	−0.013647	0.008365
9	C	−0.002151	−0.017782	−0.019933	0.015631
10	C	−0.000564	−0.002349	−0.002913	0.001785
11	C	−0.006040	−0.010958	−0.016998	0.004918
12	C	−0.001465	−0.012040	−0.013505	0.010575
13	C	−0.003252	−0.004696	−0.007948	0.001444
14	C	−0.000585	−0.003327	−0.003912	0.002742
15	C	−0.026912	−0.021708	−0.048620	−0.005204
16	H	−0.071547	−0.021041	−0.092588	−0.050506
17	H	−0.003444	−0.030455	−0.033899	0.027011
18	H	−0.110261	−0.030576	−0.140837	−0.079685
19	H	−0.077386	−0.045009	−0.122395	−0.032377
20	H	−0.001451	−0.034168	−0.035619	0.032717
21	H	0.000318	−0.021410	−0.021092	0.021728
22	H	−0.004788	−0.039328	−0.044116	0.034540
23	H	−0.095522	−0.024861	−0.120383	−0.070661
24	H	−0.092706	−0.030045	−0.122751	−0.062661
25	H	−0.001379	−0.017521	−0.018900	0.016142
26	H	−0.001192	−0.012381	−0.013573	0.011189
27	H	−0.001694	−0.013064	−0.014758	0.011370
28	H	−0.069639	−0.005376	−0.075015	−0.064263
29	H	−0.001593	−0.015722	−0.017315	0.014129
30	H	−0.001806	−0.023714	−0.025520	0.021908
31	H	−0.019843	−0.005135	−0.024978	−0.014708
32	H	−0.001495	−0.005325	−0.006820	0.003830
33	H	−0.000896	−0.005580	−0.006476	0.004684
34	H	−0.000656	−0.004908	−0.005564	0.004252
35	H	−0.000693	−0.004698	−0.005391	0.004005
36	H	−0.000543	−0.004528	−0.005071	0.003985
37	H	−0.003649	−0.018476	−0.022125	0.014827
38	H	−0.097783	−0.025521	−0.123304	−0.072262
39	H	−0.001939	−0.025914	−0.027853	0.023975

The visualization of the HOMO indicates that this molecule has a significant capacity to donate electrons. The analysis of Fukui indices is a powerful tool in investigating antioxidant molecules, as it identifies the reactive regions of the molecule, highlighting their propensity to interact with oxidizing species or free radicals. This approach aids in understanding oxidation mechanisms, predicting chemical stability, and optimizing the structure of compounds for greater efficiency in neutralizing oxidative processes, contributing to the development of new antioxidant agents [41, 50].

### 3.4 RDG and NCI Analysis

**FIGURE 6:**
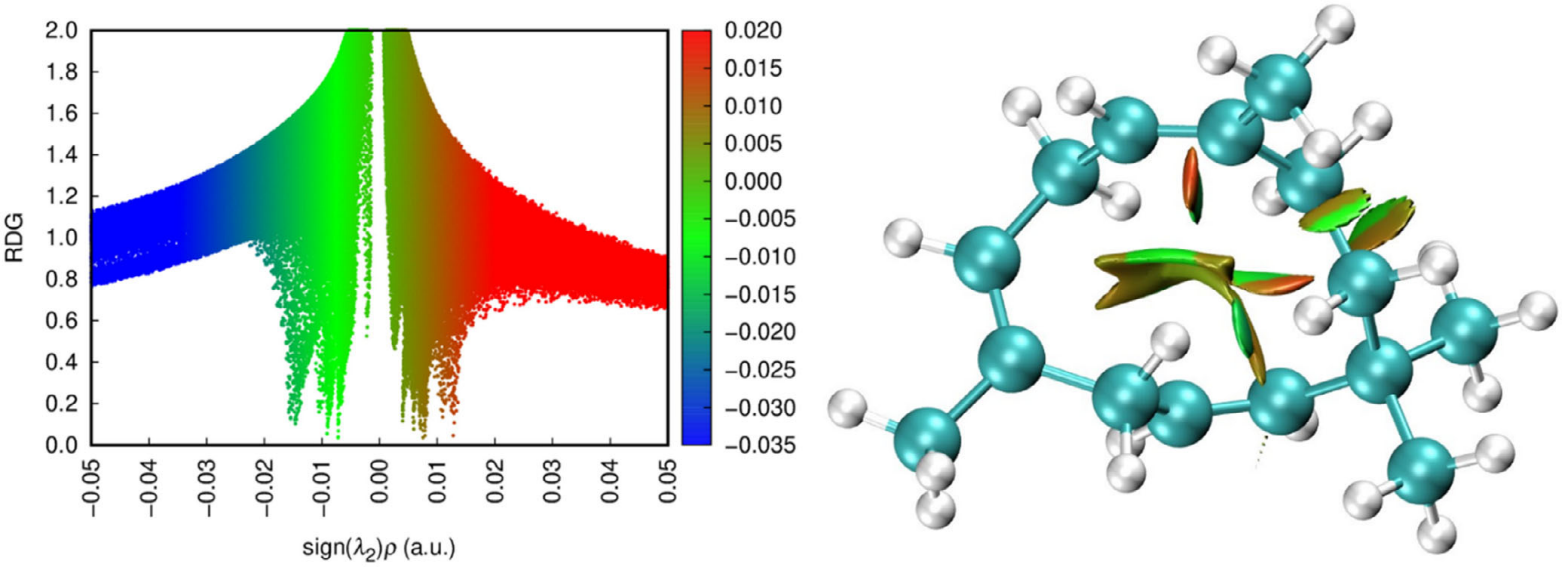
Representation of weak molecular interactions of 1,4,7‐cycloundecatriene, 1,5,9,9‐tetramethyl‐Z,Z,Z‐.

The RDG is a mathematical function that uses electron density to identify regions where the density varies smoothly, which characterizes non‐covalent interactions. It is a measure of the variation in electron density, and low RDG values indicate areas where the electron density is relatively uniform, typical of regions where weak interactions occur. The NCI method uses RDG to highlight regions of non‐covalent interactions within a molecule or between molecules. This technique identifies and characterizes weak interactions using a visual representation in graphs or colored isosurfaces (Figure 6). The intensity and type of interaction are indicated by different colors: attractive interaction regions, such as hydrogen bonds and van der Waals forces, are typically represented by blue and green colors, respectively, while repulsive interaction regions, such as steric repulsions, are represented by red tones. The NCI method is useful for visualizing and classifying non‐covalent interactions in complex systems, such as proteins, molecular aggregates, and materials. Thus, it was possible to identify regions of the molecule that are prone to chemical reactions, as these interactions can make the system energetically less favorable [51, 59].

During the observation, the focus was placed on atoms with relevance indicated by the delta Fukui index. Carbon 5C did not present non‐covalent interactions with other surrounding atoms. Carbon 2C, a nucleophilic attack region, shows slight van der Waals interactions in the most superficial part involving hydrogens H27, H28, and H18 bound to 1C. In the inner region, closer to 2C, the isosurface displayed weaker repulsive forces. The spatial volume formed between 11C and 14C exhibits similar behavior: van der Waals interactions at the level of the hydrogens, with gradually moderate steric forces deeper in the region. Between 8C and 11C, non‐covalent interactions were observed, but they were almost imperceptible.

In a cellular context, repulsive interactions can reduce molecular stability, making the molecule more susceptible to structural changes and decreasing its effectiveness in interacting with biological targets. Repulsive regions near the active site hinder recognition and binding to the target, as these forces prevent the formation of stable interactions, such as covalent or hydrogen bonds. Specifically, the absence of hydrogen bonds can limit the affinity and stability of molecular interactions, affecting the efficacy of the resulting complex. On the other hand, repulsive interactions can stabilize the molecule, making it more resistant to degradation [60].

Van der Waals forces, although individually weak, are essential for structural stability, molecular recognition, and interaction modulation in biological systems. They help compact protein structures, maintain cohesion in cell membranes, and facilitate initial interactions between molecules before stronger bonds are formed. These interactions balance cellular dynamics, allowing associations and dissociations as needed. An example is cis‐citral, which inhibits the NF‐kB p50 protein through van der Waals interactions and lipophilic contacts with residues such as Gly128 and Asp125, compensating for the absence of hydrogen bonds and stabilizing the protein‐ligand complex. This inhibition can reduce the expression of pro‐inflammatory genes, offering promise for treating inflammatory diseases and cancer [59, 61].

### 3.5 Total density of states (TDOS)

The TDOS is a function that describes the number of electronic states available per unit of energy in a system, such as a molecule, solid, or material. It is calculated by summing the contributions of all orbitals in the system and represents the distribution of electronic density across different energy levels. TDOS is important because it provides information about the system's electronic structure, helping to identify regions of higher or lower electronic density, which are directly related to chemical reactivity, optical properties, and electrical conductivity [41, 42, 58].

**FIGURE 7:**
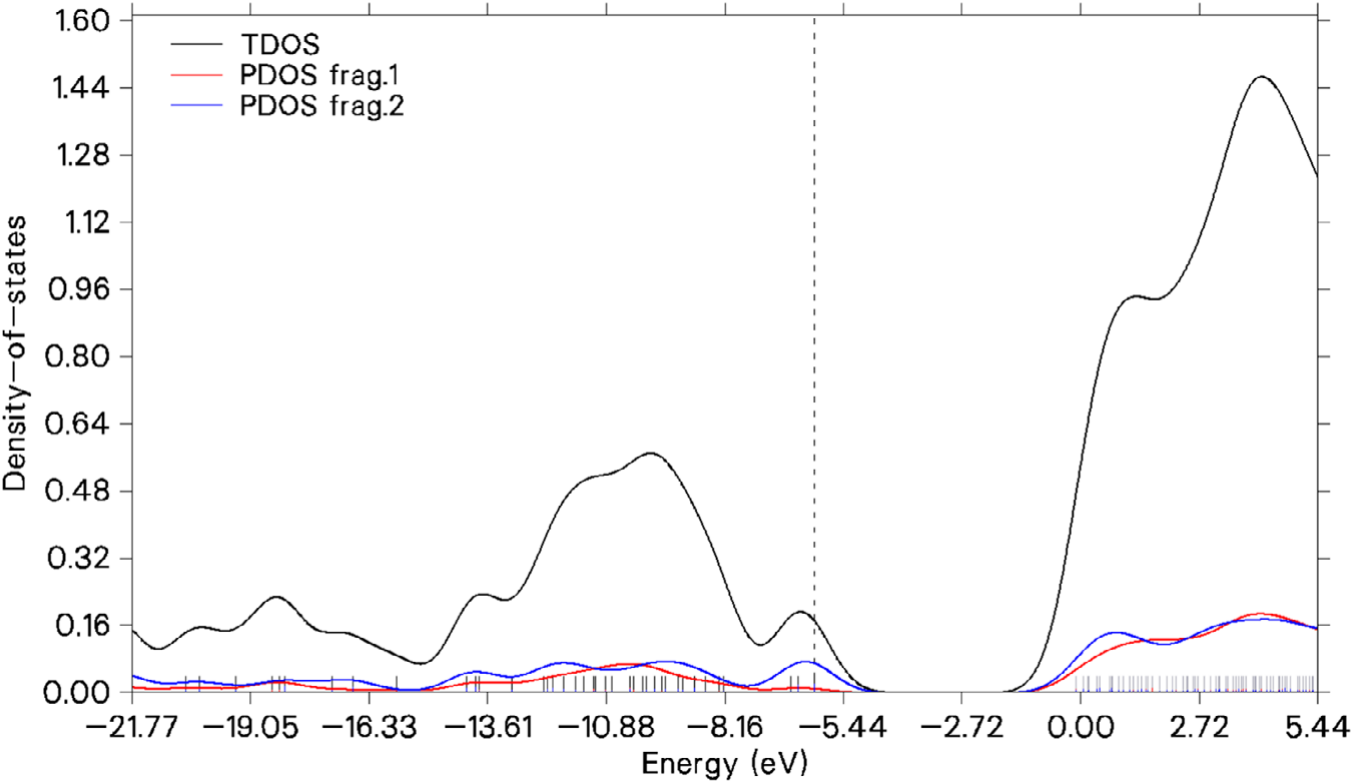
Distribution of electronic states available per unit of energy of 1,4,7‐cycloundecatriene, 1,5,9,9‐tetramethyl‐Z,Z,Z‐.

In this case, the test also serves to complement the inferences of the Fukui delta, as the visualization of the HOMO and LUMO lobes provides a spatial understanding of the probability of electronic density, while the density of states more quantitatively shows the energy distribution across orbitals. In Figure 7, the dashed line refers to HOMO. The atoms highlighted by the Fukui delta were considered as fragments to obtain the centers of the projected DOS (PDOS) individually and grouped in relation to electrophilic attack (PDOS frag 1) and nucleophilic attack (PDOS frag 2), as shown in the figure below. It is expected that regions undergoing electrophilic attacks will have LUMO with lower energy, which is evident with PDOS frag 1 (red line) in the figure, indicating low density of states near the HOMO. In comparison, molecules undergoing nucleophilic attack (blue line) have a higher density of states in the HOMO and significantly contribute to the TDOS. The analysis of the LUMO of 1,4,7‐cycloundecatriene, 1,5,9,9‐tetramethyl‐Z,Z,Z‐ suggests that its form, with extended distribution reaching outer layers, indicates potential to neutralize free radicals while maintaining good structural stability.

This stability was inferred both from the energy Gap_HOMO‐LUMO_ between frontier orbitals and from the NCI analysis, which revealed significant van der Waals and steric interactions. Additionally, ELF and LOL calculations showed the presence of delocalized electrons, suggesting an electron donor potential, as in oxidation. Based on these theoretical data, it is proposed that the molecule may act as both an oxidant and an antioxidant. This dual potential could be advantageous in specific biological contexts, such as inducing cytotoxicity through lipid peroxidation or providing cytoprotection by neutralizing reactive oxygen species, for example. It is emphasized that these interpretations are based on theoretical analyses and require experimental validation.

The TDOS and PDOS centers reflect the average position of the total and partial electronic density, respectively, providing insights into the energy distribution within the system. Calculated based on the TDOS derived from the Hirshfeld partition, the TDOS center at −2.953 eV, located above the HOMO (−6.145 eV), suggests a greater contribution from unoccupied levels. The PDOS centers indicate distinct characteristics: PDOS 1 (−1.974 eV), closer to the LUMO, represents a nucleophilic region, while PDOS 2 (−3.718 eV), closer to the HOMO, is associated with electrophilic sites. The consistent separation of fragments according to the Fukui indices validates the analysis, highlighting different roles in chemical reactivity, such as donating or accepting electronic density.

## 4 Conclusions

The enzymatic extraction of the essential oil of *M. azurea* assisted by cellulase at 1% concentration showed optimal interaction between the enzymatic action and the substrate regarding the yield, but the 1.5% concentration has better preserved the chemical profile compared to the control. It is common for essential oils to exhibit a decrease in kinetic behavior. However, the 24% increase is statistically significant, and the change in the chemical profile may offer benefits, as 1,4,7‐cycloundecatriene, 1,5,9,9‐tetramethyl‐ Z,Z,Z‐, the product of the enzymolysis reaction, has demonstrated antioxidant potential based on DFT analyses. This could enhance the performance of the essential oil in scavenging free radicals like DPPH. Oxidation is a highly relevant process for the preservation of perishables and biological reactions, such as cellular protection, cytotoxicity in tumor cells, and various aging‐related diseases. Therefore, molecules with demonstrated capacity through computational calculations are excellent candidates for experimental validation tests.

## 5 Experimental

### 5.1 Plant Material

The propagation, planting, and essential oil extraction of *M. azurea* were conducted at the Federal University of Amazonas (UFAM). The rooted cuttings were cultivated in a nursery with 50% shading in the experimental area of the Faculty of Agrarian Sciences, and planting was carried out at coordinates −3.10231, −59.97535. The planting took place in August 2022, and after the hardening off, a urea bath was applied to stimulate the increase in aerial biomass. The harvest for analysis was conducted in August 2023. Voucher specimens ‐ HUAM12737‐ were deposited in the HUAM herbarium (UFAM). The plant identification was done by Dr. Ari de Freitas Hidalgo, and specimens were collected on June 2, 2025. Climate data is available in the Supporting Information.

### 5.2 Traditional Hydrodistillation

The volatile oil content in plant material was determined by the hydrodistillation process using a Clevenger apparatus. The extractor model is designed to operate in a closed circuit and is based on hydrodynamic calculations to maintain system equilibrium throughout the extraction. The experiments were performed using 75 g of fresh material (leaves) at a mass‐to‐volume ratio of 10%. Distilled water was used as a solvent. The essential oil content extracted from plant biomass was calculated after 60 min of extraction, based on dry matter. The hydrodistillation time was determined based on kinetic evaluation, with essential oil volumes collected every 5 min during the first 60 min, followed by 10‐minute intervals for an additional 60 min. The extraction curve showed that the oil volume stabilized after approximately one hour, indicating that the majority of the extraction occurred within the first 60 min of the process.

### 5.3 Cellulase‐assisted hydrodistillation

The commercial enzyme Celluclast 1.5 L, provided by the company LFN Latino Americana, was used with a declared activity of 700 EGU/g. A moisture test was also conducted on the leaves to compensate for the water volume in the enzymatic concentration within the system. The enzyme's concentrations were: 1%, 1.5%, 2%. The enzymolysis was carried out in a 50°C bath without shaking for 30 min; the process is illustrated in Supporting Information [62]. The moisture content of the leaves was determined to remove this volume from the enzymatic concentration calculation as follows (Equation (1)):

(1) $$\%\;Moisture=\left[\left(Initial\;Mass-Dry\;Mass\right)/Initial\;Mass\right)]\times 100$$

### 5.4 Gas Chromatography/MS

The analysis was performed using a Shimadzu GC‐2030 gas chromatograph coupled to a GC‐MS‐TQ8050 NX mass spectrometer. The Shimadzu SH‐I‐5Sil MS column was used, featuring a cross‐bond stationary phase with dimensions of 30 m in length, 0.25 mm internal diameter, and 0.25 µm film thickness (df). The operational temperature range of the column used is −60°C to 320/350°C. For injection, the split mode was used with a split ratio of 10:1 and an injection volume of 0.3 µL. Pictures and the machine's setting details can be found in Supporting Information.

The oven was programmed with an initial temperature of 40°C, held for 1 min, followed by heating at a rate of 5.00°C/min up to 280°C, which was held for 11 min. The flow conditions were optimized with a pressure control set to 49.5 kPa, a total flow of 14.0 mL/min, a column flow of 1.00 mL/min, and a linear velocity of 36.1 cm/s. A purge flow of 3.0 mL/min was employed to maintain sample efficiency.

MS conditions included an ion source temperature of 230°C and an interface temperature of 250°C. The solvent cut time was set to 3.0 min, and acquisition was performed in Q3 Scan mode, with a scan range of 20 to 600 *m/z* and a scan speed of 2000 amu/s. Data was collected with a gain relative to the tuning adjustment, set at 0.98 kV.

The identification of the sample components was based on the fragmentation patterns observed in the mass spectra, compared with existing values in the National Institute of Standards and Technology databases.

### 5.5 Antioxidant Activity (DPPH)

The method involves evaluating the scavenging capacity of the free radical DPPH, which has a purple color and a maximum absorption at 517 nm. Upon interaction with an antioxidant (AH) or a radical species (R), DPPH· is reduced, forming diphenyl‐picrylhydrazine, which is yellow in color, leading to a decrease in absorption that can be monitored by the reduction in absorbance. From the results obtained, the percentage of antioxidant activity or free radical scavenging activity, and/or the percentage of remaining DPPH· in the reaction medium, is determined.

The DPPH solution was prepared at a concentration of 0.06 mol/L, with the sample weighed and diluted with methanol P.A. to 1000 mL. The solution was prepared on the same day as the analysis and protected from light exposure. Aliquots of 50 µL of each test sample concentration were transferred in triplicate to test tubes protected from direct light exposure, followed by the addition of 1950 µL of the 0.06 mol/L DPPH solution and mixing. The control was prepared similarly, replacing the sample with methanol P.A. The blank was prepared by adding 1950 µL of methanol P.A. plus 50 µL of the test solution at the same concentration as each assay to calibrate the spectrophotometer. After 30 min of reaction, absorbance was measured using a spectrophotometer at a wavelength of 517 nm [63]. The DPPH inhibition was calculated as follows (Equation (2)):

(2) $$\begin{array}{ccc} \%\;inhibition & = & [\left(Abs\;control-Abs\;sample\right)/\left(Abs\;control)\right]\\ & & \times 100 \end{array}$$

The data were expressed as means and standard deviations and subjected to Student's t‐test for comparing two means and One‐Way ANOVA for variance analysis between more than two means. Differences with *p* ≤ 0.05 were considered significant.

### 5.6 Computational Analysis by DFT

The functional B3LYP (Becke, 3‐parameter, Lee‐Yang‐Parr) with the 6‐311++G(d,p) basis set was selected for a balance between computational cost and precision. DFT is a combination of methods that approximates the calculation of a molecule's electronic energy while considering quantum mechanics effects. Here, “B” refers to the Becke exchange functional, a correction to the Hartree‐Fock exchange functional. The “3LYP” combines three functionals: 1) Hartree‐Fock exchange, 2) Becke exchange (B), and 3) Lee‐Yang‐Parr correlation (LYP) (Becke, 1993). Calculations were performed simulating the gaseous state and implicit solvation in aqueous medium using CPCM (water). The B3LYP‐D3BJ functional was employed, which incorporates Grimme's D3BJ dispersion corrections to improve the accuracy of non‐covalent interactions. Geometry optimizations (OPT) were carried out with TightSCF convergence criteria to achieve high numerical precision. The Hirshfeld partitioning method was applied to analyze atomic contributions to the electronic density. KDIIS and SOSCF algorithms were used to enhance the self‐consistent field (SCF) convergence, ensuring stability in electronic structure calculations. Additionally, PAL6 parallelization was implemented to improve computational efficiency.

To obtain Fukui indices [16], atomic charges were calculated using the Hirshfeld method. The Cartesian coordinates calculated after molecule optimization are available in the Supporting Information. The following quantum descriptors were calculated: IP, EA, Gap_HOMO‐LUMO_ (ΔE), Electronegativity (χ), Global Hardness (η), Global Softness (S), Chemical Potential (μ), Electrophilicity Index (ω), Maximum Charge Transfer Index (ΔN_max_), and Nucleophilicity Index (N) [64].

The quantum descriptors were calculated using Equations (2)–(11):

(2) $$\mathrm{IP}=-E_{\mathrm{HOMO}}$$

(3) $$\mathrm{EA}=-E_{\mathrm{LUMO}}$$

(4) $$\mathrm{Ga}p_{\mathrm{HOMO}-\mathrm{LUMO}}\left(\Delta E\right)=\mathrm{IP}-\mathrm{EA}$$

(5) $$\chi =1/2\left(\mathrm{EA}+\mathrm{IP}\right)$$

(6) $$\eta =1/2\left(\mathrm{IP}-\mathrm{EA}\right)$$

(7) S=1/η

(8) $$\mu =-1/2\left(\mathrm{EA}+\mathrm{IP}\right)$$

(9) $$\omega =\mu^{2}/2\eta$$

(10) $$\Delta N_{\max}=-\mu /\eta$$

(11) N=1/ω

### 5.7 Software

The statistical analysis was performed using Origin 2018; DFT calculations were conducted with Orca 6.0; input files for ELF, LOL, MEP, RDG, and NCI analyses were generated in Multiwfn. Visualization of frontier orbitals was performed with Iboview; RDG with Irfanview; NCI and MEP analysis with VMD 1.9.3 [65, 66, 67, 68].

## Author Contributions

**Júlio César Gonçalves de Souza**: Writing and editing, conceptualization, data curation, investigation, laboratory analyses, production of feedstock. **Leiliane do Socorro Sodré de Souza**: conceptualization, data curation, investigation, review. **Ari de Freitas Hidalgo**: production of feedstock. **Guilherme Teixeira de Azevedo**: laboratory analyses. **Giovana Lima de Souza**: laboratory analyses. **Caroline Dutra Lacerda**: laboratory analyses. **Sergio Duvoisin Junior**: laboratory analyses. **Anderson Mathias Pereira**: conceptualization, data curation, investigation, review.

## Conflicts of Interest

The authors declare no conflicts of interest.

## Supporting information




**Supporting File 1**: cbdv70340‐sup‐0001‐SuppMat.pdf

## Data Availability

The authors have nothing to report.
