# Supplementary material for: DPPH‐based Antioxidant Screening and Cellulase‐assisted Hydrodistillation of Matourea azurea: Improved Essential Oil Yield and Density Functional Theory Insights Into a Rare Terpene
Source: Chem Biodivers. 2025 Aug 7;22(12):e01109. doi: 10.1002/cbdv.202501109 (PMC12715992; doi:10.1002/cbdv.202501109)
Supplement: Supplementary file 1 — Supporting File 1: cbdv70340‐sup‐0001‐SuppMat.pdf [file CBDV-22-e01109-s001.pdf]

Supporting Information

Antioxidant activity and essential oil extraction of *M. azurea* by cellulase-assisted hydrodistillation: DFT analysis of 1,4,7-cycloundecatriene, 1,5,9,9-tetramethyl- Z,Z,Z, a product of Enzymolysis

Júlio César Gonçalves de Souza[a] Leiliane do Socorro Sodré de Souza[a], Ari de Freitas Hidalgo[b], Guilherme Teixeira de Azevedo[c], Giovana Lima de Souza[c], Caroline Dutra Lacerda[d], Sergio Duvoisin Junior[d] and Anderson Mathias Pereira*[,a]

[a] Graduate Program in Environmental Sciences and Sustainability in the Amazon (PPG-CASA), Federal University of Amazonas, andersonpereira@ufam.edu.br

[b] Department of Animal and Plant Production, Federal University of Amazonas

[c] Department of Chemical Engineering and Food Engineering, Federal University of Santa Catarina

[d] Applied Chemistry Research Group for Technology, State University of Amazonas

---

Table of Contents

Figure S1. *M. azurea* antioxidant activity (DPPH).

Figure S2. Map of electrostatic potential with isovalue of 0.002 a.u.

Table S1. Climate Data of Manaus (AM), Brazil in 2022 – 2023.

Figure S3. Graphical cellulase-assisted hydrodistillation method.

Figure S4. Shimadzu GC-2030 gas chromatograph coupled to a GCMS-TQ8050 NX mass spectrometer.

Table S2. Molecule optimization at B3LYP/6-311++G (d,p), CPCM(Water).

Antioxidant Activity (DPPH)

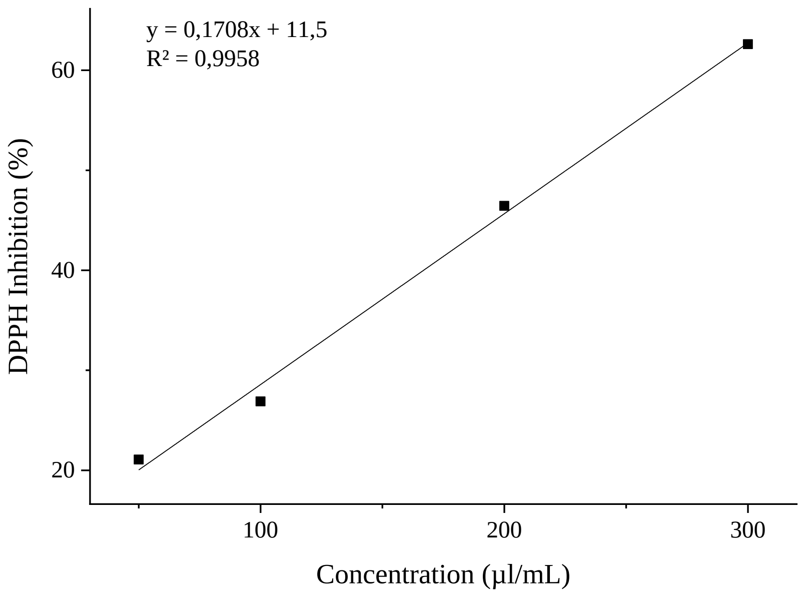

Figure S1. *M. azurea* antioxidant activity (DPPH).

Mapping of Electrostatic Potential

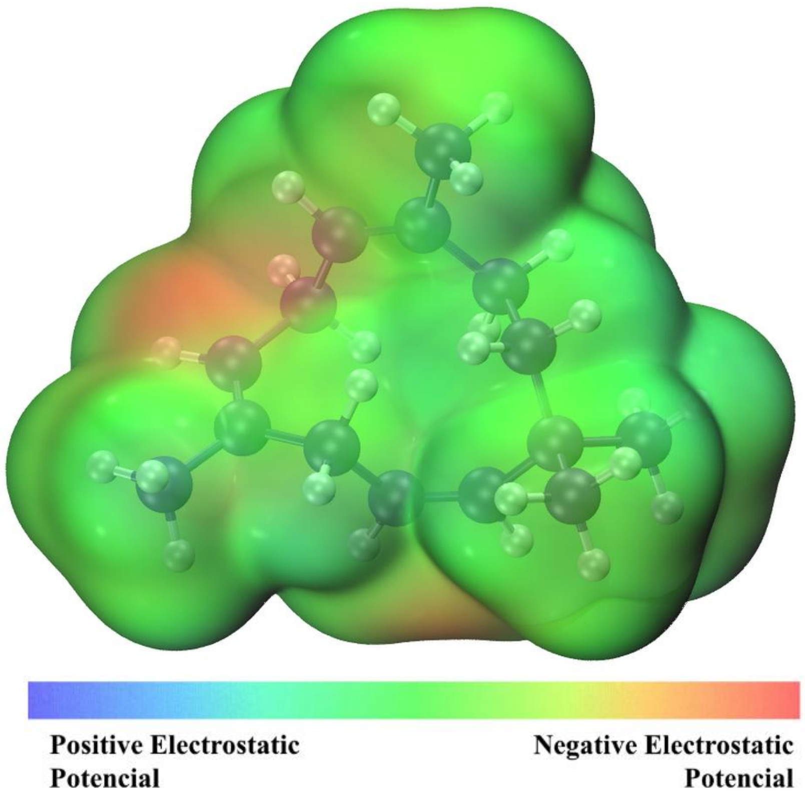

Figure S2. Map of electrostatic potential of 1,4,7-cycloundecatriene, 1,5,9,9-tetramethyl- Z,Z,Z, with isovalue of 0.002 a.u.

Climate Data (Plant Material)

We chose to provide data on the main climatic variables during the planting and harvesting of *M. azurea*, as we believe it may be useful for researchers working with mathematical modeling to investigate how climate qualitatively affects the chemical profile of essential oils.

Table S1. Climate Data of Manaus (AM), Brazil in 2022 – 2023.

**Year**	**Month**	**Total Precipitation (mm)**	**Maximus Temperature (ºC)**	**Umidity (%)**	**Global Radiation (Kj/m²)**
2022	January	0.425981055	27.30270636	80.73612991	558.0434371
2022	February	0.461921708	27.01921708	82.39288256	531.2886833
2022	March	0.48117702	26.83110696	83.37505838	522.5647828
2022	April	0.514195804	26.84751748	83.5513986	519.1403846
2022	May	0.443087174	27.01488062	82.44031094	529.2707385
2022	June	0.403750868	27.05262792	81.79022922	536.5004168
2022	July	0.350465254	27.3571372	80.02989507	555.4585627
2022	August	0.311770797	27.6365378	78.32550915	576.3720228
2022	September	0.278869779	27.94609951	76.80113636	592.1259367
2022	October	0.272060648	28.08843556	76.12074431	602.2450861
2022	November	0.259902047	28.15614167	75.75709621	606.6010800
2022	December	0.281495112	28.13659995	76.21900161	607.0407936
2023	January	0.374068966	26.43972414	86.67724138	515.1800000
2023	February	0.453740015	26.46928105	87.26724764	526.6848947
2023	March	0.490995261	26.67279621	87.02748815	528.9925592
2023	April	0.464962726	26.90134943	86.44389205	537.0014554
2023	May	0.402339181	27.22435219	85.63137364	532.1568042
2023	June	0.349613674	27.43445772	84.40056219	521.7931317
2023	July	0.311665005	27.43445772	84.40056219	521.7931317
2023	August	0.274526828	28.23800591	80.37675873	543.9511992
2023	September	0.250818659	28.58671199	78.70720025	563.6922399
2023	October	0.237890354	28.87240039	77.41149521	577.5032222
2023	November	0.233552299	29.01432904	76.92330048	583.7832512
2023	December	0.251250579	28.93919388	77.58744498	576.5646623

Cellulase-assisted hydrodistillation

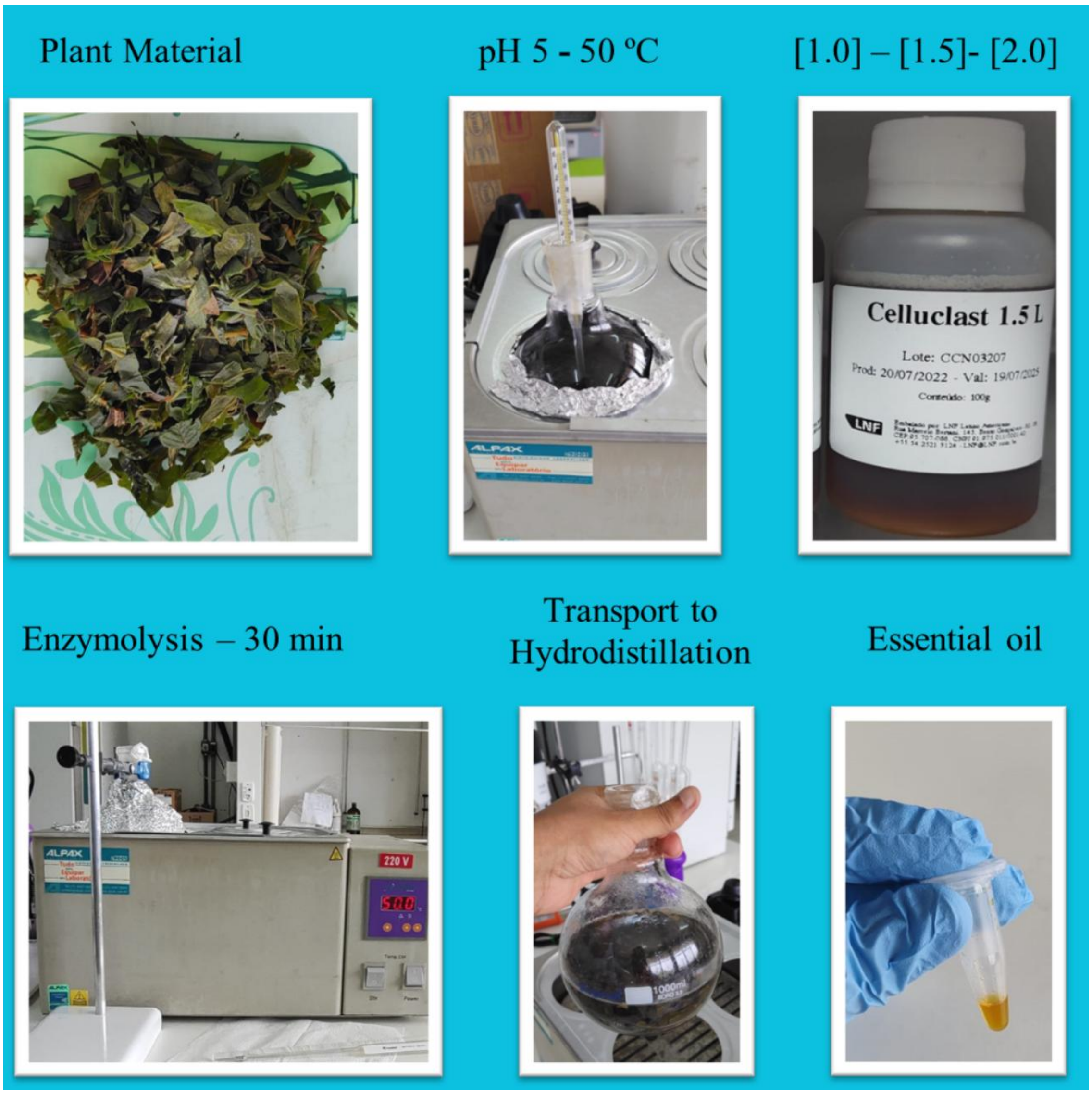

Figure S3. Graphical cellulase-assisted hydrodistillation method.

Gas Chromatography/Mass Spectrometry (GC/MS)

Equipment settings: In the GC-2030 system. The conditions include a column oven temperature set at 40.0 °C. with an injection temperature of 250.0 °C and the injection mode configured as Split. The flow control mode operates under pressure. set at 49.5 kPa. with a total flow of 14.0 mL/min and a column flow of 1.00 mL/min. resulting in a linear velocity of 36.1 cm/s. The purge flow is set at 3.0 mL/min. with a split ratio of 10.0. Features such as high-pressure injection. carrier gas saver. and splitter hold are deactivated. The oven program follows an initial heating to 40.0 °C with a hold time of 1.00 min. followed by an increase to 280.0 °C with a hold time of 11.00 min. The oven cooling rate is set to Middle.
In the readiness checks. the heating of units includes the activation of the column oven. SPL-AOC. and MS. Other flow checks indicate that the SPL-AOC carrier and SPL-AOC purge flows are active. The conditions also include an equilibrium time of 3.0 minutes. while the auto-flame controls are set with auto-flame off as ON and re-ignite and threshold remain deactivated. In the GCMS-TQ8050 NX system. The ion source temperature is set to 230.0 °C. with the interface temperature also at 250.0 °C. The solvent cut time is defined as 3.0 minutes. and the detector gain mode is configured relative to the tuning result. The detector voltage is set to 0.98 kV ± 0.00 kV. with the threshold set to 0. Data acquisition without using CID Gas (Q3Scan) is activated (ON).

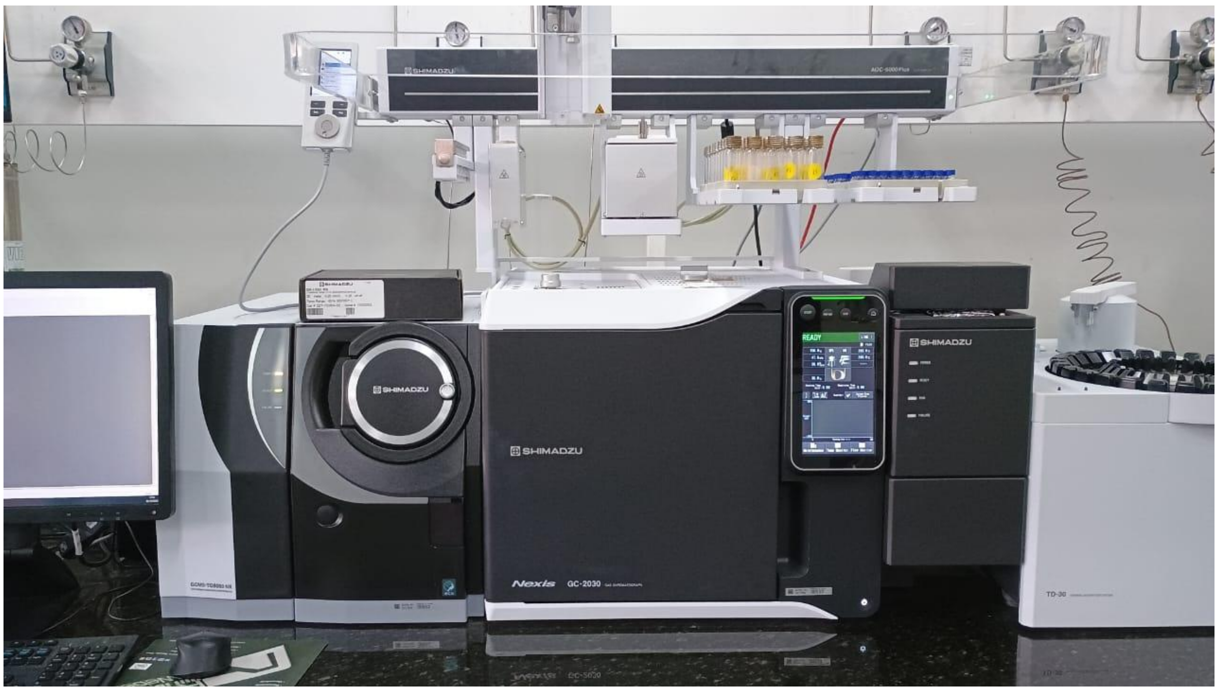

Figure S4. Shimadzu GC-2030 gas chromatograph coupled to a GCMS-TQ8050 NX mass spectrometer.

Computational Analysis by DFT

Table S2. Molecule optimization at B3LYP/6-311++G (d.p). CPCM(Water).

	Cartesian Coordinates (Angstroem)								
	Neutral			Cation			Anion		
Atom	x	y	z	x	y	z	x	y	z
C	9.835361	2.132471	3.668006	9.671104	2.418236	3.638410	9.885727	2.114744	3.631430
C	8.940109	2.319872	2.467878	8.880210	2.421564	2.375432	8.951958	2.325213	2.467298
C	7.775948	2.976885	2.571043	7.690365	3.128550	2.315733	7.790638	2.977832	2.623178
C	6.763803	3.237261	1.479672	6.746339	3.217047	1.159762	6.752386	3.273419	1.566511
C	5.400472	2.629976	1.757243	5.664467	2.424283	1.840451	5.390856	2.641273	1.803511
C	5.101960	1.325319	1.709969	5.305900	1.102185	1.717401	5.105322	1.335394	1.713667
C	6.139880	0.264294	1.382916	5.903625	0.050216	0.824757	6.170538	0.297098	1.416450
C	6.514202	0.228510	-0.080851	6.594716	0.452053	-0.447168	6.502073	0.197799	-0.056534
C	7.678850	-0.080069	-0.663313	7.843432	0.174402	-0.835911	7.656002	-0.129086	-0.651666
C	9.017294	-0.538537	-0.090743	8.970212	-0.507022	-0.065564	9.007441	-0.550629	-0.082490
C	9.433042	0.142418	1.234252	9.188861	0.155641	1.316859	9.412114	0.151401	1.232807
C	9.445487	1.691947	1.189875	9.449725	1.688507	1.211525	9.405836	1.698918	1.169651
C	8.932675	-2.061346	0.154462	8.658691	-2.005517	0.144427	8.953998	-2.071833	0.184478
C	10.105775	-0.265985	-1.149284	10.264915	-0.389834	-0.891803	10.085752	-0.271995	-1.149402
C	3.706783	0.821320	1.969729	4.164517	0.607115	2.551276	3.696762	0.822244	1.856728
H	9.413896	2.594220	4.563716	9.178184	2.958611	4.445979	9.497120	2.563649	4.547548
H	10.824369	2.568915	3.483304	10.663374	2.848196	3.459032	10.867757	2.555905	3.419483
H	9.997517	1.068276	3.874089	9.844957	1.381177	3.953277	10.054976	1.046000	3.808704
H	7.499062	3.366286	3.550297	7.388640	3.666383	3.209588	7.552059	3.342433	3.621402
H	6.636918	4.320411	1.371916	6.418734	4.240793	0.975881	6.611903	4.359485	1.521313
H	7.137432	2.873007	0.520665	7.120398	2.766096	0.248153	7.120696	2.971664	0.583149
H	4.603071	3.326564	2.008187	5.125454	2.987290	2.596547	4.574205	3.326235	2.021991
H	5.734482	-0.717008	1.658847	5.075034	-0.616155	0.548610	5.823373	-0.687144	1.757696
H	7.016101	0.418637	2.008053	6.537292	-0.571644	1.463068	7.058120	0.522352	1.997441
H	5.690605	0.470806	-0.751405	5.957077	0.980218	-1.152648	5.661247	0.401026	-0.718645
H	7.668982	-0.065065	-1.752667	8.110174	0.495123	-1.840610	7.625309	-0.166002	-1.740286
H	10.429655	-0.231336	1.490360	10.037451	-0.323274	1.812451	10.412214	-0.204176	1.500148
H	8.779117	-0.202219	2.037213	8.323516	-0.024001	1.954777	8.756681	-0.189975	2.036604
H	8.849417	2.028906	0.342664	9.017491	2.062578	0.284483	8.766470	2.020147	0.348153
H	10.468512	2.039357	1.006938	10.526604	1.869136	1.174237	10.414713	2.056893	0.934802
H	8.176450	-2.287466	0.911258	7.764504	-2.156197	0.752547	8.202500	-2.302229	0.944590
H	9.897293	-2.446096	0.502457	9.498387	-2.494576	0.647438	9.926224	-2.429811	0.539936
H	8.665982	-2.590461	-0.765109	8.501360	-2.498516	-0.818423	8.699849	-2.620992	-0.726845

H	10.189771	0.802603	-1.366746	10.519311	0.654112	-1.093656	10.149912	0.794841	-1.381299
H	9.873123	-0.782253	-2.085547	1.0150742	-0.898121	-1.853509	9.859774	-0.805262	-2.077780
H	11.078559	-0.619590	-0.795227	11.100534	-0.849163	-0.356778	11.066188	-0.603265	-0.794541
H	3.012069	1.636973	2.182602	3.851993	1.330333	3.303633	2.991419	1.628439	2.071363
H	3.697942	0.124097	2.816197	4.445380	-0.332613	3.041243	3.622253	0.065070	2.646662
H	3.332168	0.262710	1.103094	3.310462	0.371400	1.905100	3.381766	0.327536	0.928804
